# Mice null for the deubiquitinase USP18 spontaneously develop leiomyosarcomas

**DOI:** 10.1186/s12885-015-1883-8

**Published:** 2015-11-10

**Authors:** Fadzai Chinyengetere, David J. Sekula, Yun Lu, Andrew J. Giustini, Aarti Sanglikar, Masanori Kawakami, Tian Ma, Sandra S. Burkett, Burton L. Eisenberg, Wendy A. Wells, Paul J. Hoopes, Elizabeth G. Demicco, Alexander J Lazar, Keila E. Torres, Vincent Memoli, Sarah J. Freemantle, Ethan Dmitrovsky

**Affiliations:** 1 Department of Pharmacology and Toxicology, Dartmouth, Hanover, NH USA; 2 Department of Medicine, Dartmouth, Hanover, NH USA; 3 Department of Pathology, Dartmouth, Hanover, NH USA; 4 Department of Surgery, Dartmouth, Hanover, NH USA; 5 Norris Cotton Cancer Center, Lebanon, NH USA; 6 Geisel School of Medicine, Dartmouth, Hanover, NH USA; 7 Dartmouth-Hitchcock Medical Center, Lebanon, NH USA; 8 Thayer School of Engineering, Dartmouth College, Hanover, NH USA; 9 Comparative Molecular Cytogenetics Core, Mouse Cancer Genetics Program, National Cancer Institute, Frederick, MD USA; 10 Department of Pathology, The Mount Sinai Hospital, New York, NY USA; 11 Department of Pathology, The University of Texas MD Anderson Cancer Center, Houston, TX USA; 12 Sarcoma Research Center, The University of Texas MD Anderson Cancer Center, Houston, TX USA; 13 Department of Surgical Oncology, The University of Texas MD Anderson Cancer Center, Houston, TX USA; 14 Present address: MD Anderson Cancer Center, Houston, TX 77030-4009 USA

**Keywords:** USP18, ISG15, Leiomyosarcoma, Murine cancer model

## Abstract

**Background:**

USP18 (ubiquitin-specific protease 18) removes ubiquitin-like modifier interferon stimulated gene 15 (ISG15) from conjugated proteins. USP18 null mice in a FVB/N background develop tumors as early as 2 months of age. These tumors are leiomyosarcomas and thus represent a new murine model for this disease.

**Methods:**

Heterozygous USP18 +/− FVB/N mice were bred to generate wild-type, heterozygous and homozygous cohorts. Tumors were characterized immunohistochemically and two cell lines were derived from independent tumors. Cell lines were karyotyped and their responses to restoration of USP18 activity assessed. Drug testing and tumorigenic assays were also performed. USP18 immunohistochemical staining in a large series of human leiomyosacomas was examined.

**Results:**

USP18 −/− FVB/N mice spontaneously develop tumors predominantly on the back of the neck with most tumors evident between 6–12 months (80 % penetrance). Immunohistochemical characterization of the tumors confirmed they were leiomyosarcomas, which originate from smooth muscle. Restoration of USP18 activity in sarcoma-derived cell lines did not reduce anchorage dependent or independent growth or xenograft tumor formation demonstrating that these cells no longer require USP18 suppression for tumorigenesis. Karyotyping revealed that both tumor-derived cell lines were aneuploid with extra copies of chromosomes 3 and 15. Chromosome 15 contains the *Myc* locus and *MYC* is also amplified in human leiomyosarcomas. MYC protein levels were elevated in both murine leiomyosarcoma cell lines. Stabilized P53 protein was detected in a subset of these murine tumors, another feature of human leiomyosarcomas. Immunohistochemical analyses of USP18 in human leiomyosarcomas revealed a range of staining intensities with the highest USP18 expression in normal vascular smooth muscle. USP18 tissue array analysis of primary leiomyosarcomas from 89 patients with a clinical database revealed cases with reduced USP18 levels had a significantly decreased time to metastasis (*P* = 0.0441).

**Conclusions:**

USP18 null mice develop leiomyosarcoma recapitulating key features of clinical leiomyosarcomas and patients with reduced-USP18 tumor levels have an unfavorable outcome. USP18 null mice and the derived cell lines represent clinically-relevant models of leiomyosarcoma and can provide insights into both leiomyosarcoma biology and therapy.

**Electronic supplementary material:**

The online version of this article (doi:10.1186/s12885-015-1883-8) contains supplementary material, which is available to authorized users.

## Background

Soft tissue sarcomas are heterogeneous malignancies of mesenchymal origin. They arise from supporting tissues of the body including adipose, muscle and fibrous connective tissue [1]. Although relatively rare, with an international incidence ranging from 1.8 to 5 cases per 100,000 per year, approximately 50 % of patients succumb to this malignancy [2–4]. Median survival for patients with advanced or metastatic sarcoma is only 12 months [5–7]. There are currently limited therapeutic options for those diagnosed with recurrent or advanced stage sarcoma. Surgery and radiation are the mainstays of treatment with doxorubicin being the most frequently used single chemotherapeutic agent, providing only 10–30 % objective responses, but no increase in survival [8, 9]. Hence, there is a need to identify new therapeutic approaches for these diverse tumors.

Leiomyosarcomas arise from smooth muscle and belong to the subgroup of sarcomas that have a complex, unbalanced karyotype, usually harboring non-specific genetic alterations and chromosomal instability [10, 11]. Consistent with other sarcomas with complex karyotypes, leiomyosarcomas frequently deregulate P53 expression [12–14]. Effective targeted therapy for the complex karyotype-associated sarcomas is likely hindered by their tumor heterogeneity. If there were a way to identify subsets of sarcomas with specific genetic alterations, this would improve the classification or treatment outcomes for these malignancies. For instance, polysomy of chromosome 8 in humans (syntenic to polysomy 15 in mice) is reported in complex sarcomas including leiomyosarcomas [15, 16]. Notably, *MYC* is found on chromosome 8 and is a frequently overexpressed oncogene in human cancers. Indeed, MYC overexpression in a subset of leiomyosarcomas was associated with decreased metastasis-free survival [17]. There are few tractable mouse models that mimic the histopathology and molecular characteristics of human leiomyosarcomas. New mouse models could be instrumental in therapeutic development.

Ubiquitin-specific protease 18 (USP18) is a deubiquitinase for an interferon-regulated ubiquitin-like process, ISGylation [18]. This is the conjugation of interferon (IFN)-stimulated gene 15 (ISG15), a 15kDa ubiquitin-like moiety, to diverse target proteins [19, 20]. USP18 knockout mice were initially generated on a C57Bl/6 and 129 mixed background [21]. These mice displayed neurological symptoms and hydrocephalus and did not survive beyond 5 months [21]. Subsequent generation of USP18 knockout mice on a pure C57Bl/6 background found homozygous deletion of USP18 was embryonic lethal [22]. In contrast, this phenotype was not seen in the USP18 knockout FVB background mice. C57Bl/6-129-USP18 knockout mice were also hypersensitive to type I IFN as seen when they were treated with the IFN-inducer, poly-IC; this was fatal for USP18 knockout, but not for the wild-type mice [23]. Recently, human ISG15 deficiency was found to cause a decrease in USP18 accumulation and this was hypothesized to cause the loss of negative feedback of type I interferon signaling in these patients leading to auto-inflammation [24].

USP18 overexpression is associated with augmented oncogene or growth factor receptor expression such as the epidermal growth factor receptor (EGFR) and tumor-promoting effects in acute promyelocytic leukemia, kidney and lung cancer [25–28]. Intriguingly, we report here the previously unrecognized development of spontaneous subcutaneous sarcomas, histopathologically diagnosed as leiomyosarcoma, in FVB-USP18 knockout mice. These murine sarcomas recapitulate critical characteristics of human leiomyosarcoma including aneuploidy, overexpression of MYC and deregulation of P53.

USP18 analysis of clinical leiomyosarcoma revealed abundant staining in normal smooth muscle cells, which was retained by some sarcomas, but lost in others. While loss of USP18 did not significantly impact overall survival or disease-free survival, it did significantly decrease the time to metastasis indicating a key role for USP18 levels in leiomyosarcoma clinical biology.

Cell lines were independently derived from sarcomas that arose in different mice. These sarcomas were histopathologically diagnosed as leiomyosarcomas. Cell lines were aneuploid and overexpressed MYC relative to mouse embryonic fibroblasts (MEFs). These lines formed rapidly growing subcutaneous sarcomas following transplantation into athymic and immunocompetent mice. These murine sarcoma cell lines, when coupled with the parental USP18 null mice, comprise tractable models to accelerate the discovery and development of new therapies for human leiomyosarcoma.

## Methods

### Mice

FVB-USP18 heterozygous mice were purchased from the Jackson Laboratory. These mice were bred to generate FVB-USP18 knockout mice. Generation of FVB-USP18 knockout mice is previously described [21, 29]. Genotyping for the knockout was done using polymerase chain reaction (PCR) assays according to a previously published protocol from the Jackson Laboratory (#007225). Mice were bred in a non-pathogen environment, according to an Institutional Animal Care and Use Committee (IACUC)-approved protocol at Dartmouth. All mice experiments were done in accordance with this protocol. Animals are housed in cages with a 68 inch squared area with no more than 4 adult mice per cage. They live with a 12 h light/12 h dark light schedule at a humidity of 30–70 % and a temperature of 72 +/− 2 ^0^ F. Mice receive ad libitum food (Teklad Irradiated Rodent Diet 2918) and water. Bedding is hardwood chip and at a depth of 0.25 inches.

Once tumors were detected in USP18 null mice, animals were closely monitored three times per week and weighed. Mice with greater than 10% body weight loss or with a tumor that was causing substantial clinical symptoms were euthanized using IACUC-approved procedures.

### Tumor tissues

Acquisition of tissue specimens and clinical information and subsequent analyses were approved by the Institutional Review Board (IRB) of The University of Texas MD Anderson Cancer Center (UTMDACC) and Dartmouth Hitchcock Medical Canter (DHMC). Patients provided written informed consent.

### Generation of USP18 knockout cell lines and cell culture

Two independent cell lines (designated KHC-1and KHC-2) were generated from subcutaneous tumors, using described methods [30]. Cells were cultured on tissue culture plates in RPMI-1640 media with 10 % fetal bovine serum (FBS). These cells have been continuously cultured for up to 40 passages at 5 % CO_2_ and at 37° C in a humidified incubator. Cell karyotyping analysis confirmed these cells were of murine origin and immunohistochemical analysis established they retained expression of antigens consistent with the original tumors. SK-LMS-1 human leiomyosarcoma cells were purchased from ATCC where cell identity is confirmed using standard short tandem repeat (STR) analyses. SK-LMS-1 cells were cultured in Eagle’s Minimum Essential medium (EMEM).

The siRNAs for knockdown experiments were from Ambion. MYC siRNA #1 (#s70224), MYC siRNA #2 (#s70226) and negative control (#4390843) were individually transfected into cells using Lipofectamine 2000 (Invitrogen) according to the manufacturer’s instructions and with triplicate replicate transfections. Protein was collected and analyzed by immunoblot assays to verify knockdown 24 h post-transfection. To quantify MYC protein knockdown, densitometry readings of immunoblots (from 3 independent transfections) were measured using NIH ImageJ analyses. Cell growth was assessed 72 h post-transfection using the CellTiter-Glo assay (Promega). Growth comparisons were normalized to cells transfected with control siRNA.

### Reconstitution of USP18 in sarcoma cell lines

Lipofectamine 2000 (Invitrogen) was used to transfect the pRetroX-IRES-ZsGreen1-USP18 retroviral vector or an empty retrovirus (Clontech) into Plat-A retroviral packaging cells according to manufacturer’s protocol (Cell Biolabs). Viral supernatants were collected after 48 h. KHC-1 and KHC-2 cells were transduced with the respective viral supernatants in regular culture media supplemented with polybrene (4g/mL) (Sigma). Green fluorescent protein expressing cells were sorted after 48 h using a FACStar Plus cytometer (Becton Dickinson).

### Drug treatments, proliferation and apoptosis assays

KHC-1, KHC-2 and human leiomyosarcoma SK-LMS-1 cells were each treated with doxorubicin (0.2μM) or with interferon-beta (IFNβ) (500 units/mL) (Sigma). Cell growth was assayed using a CellTiter-Glo Luminescent assay (Promega) in four independent, replicate experiments (each performed in at least triplicates). Unless stated otherwise, growth was measured after 72 h. Apoptosis was analyzed using Annexin V:FITC and PI positivity in flow cytometry assays according to the manufacturer’s instructions (AbD Serotec). All experiments were performed at least 3 times.

### Immunoblot analyses

Cells were lysed in modified radioimmunoprecipation buffer (RIPA) [31] supplemented with protease and phosphatase inhibitors (Sigma). Proteins were resolved on SDS-PAGE gels and transferred to nitrocellulose membranes (primary antibodies listed in Additional file 1: Table S1). Membranes were blocked in tris-buffered saline with 0.1 % Tween 20 (TBS/Tween) plus 5 % non-fat milk powder. Primary antibodies were diluted in this blocking buffer or in 5 % bovine serum albumin (BSA) in TBS/Tween. Primary antibodies were detected with horseradish peroxidase (HRP) conjugated secondary antibodies (Santa Cruz and GE Healthcare) and visualized using Luminata Forte chemiluminescent HRP detection reagents (Millipore).

### Anchorage independent growth

Cells were plated in 6 well plates (10^4^ cells/well) in 0.5 % agarose, as before [31]. The agar and cell suspensions were layered onto a 1 % base agarose in RPMI-1640 media containing 10% FBS. After formation of the top agar, 1mL of RPMI-1640 medium containing 10% FBS was overlaid before incubation at 37°C in a 5 % CO_2_humidified incubator for 14 days. Colonies greater than five cells were counted using a microscope in four independent experiments.

### Orthotopic sarcoma model

Cells for subcutaneous flank implantation into nude mice were resuspended in a 1:1 mixture of phosphate buffered saline (PBS) and reduced growth factor Matrigel (BD Biosciences). The cell number used for athymic nude mice (01B74/Athymic NCr-nu/nu, NCI) was 2.7 × 10^5^ for the KHC-1 line, 1 × 10^5^ for KHC-2 cells and for syngeneic NCr-FVB (NCI) mice this was 1 × 10^5^ KHC-1 cells. Four 8 week old female mice were used per cell line for subcutaneous tumor growth analysis and were randomly assigned the different cell lines. Mice were weighed and tumor size was measured biweekly with calipers. The orthotopic mouse tumor experimental protocol was reviewed and approved by Dartmouth's Institutional Animal Care and Use Committee (IACUC). Mice were sacrificed using an IACUC-approved protocol when tumor diameter measured 15 mm or if mice became symptomatic and/or cachectic (loss of 10 % or more of body weight). Tumor volume was calculated using the formula 0.5 × length × (width)^2^ where width is defined as the smallest diameter.

### Histopatology

Routine hematoxylin and eosin (H & E) and immunostaining were performed as described in the Additional file 2.

### Statistics

Two-tailed t tests were used for statistical analysis unless otherwise noted. Statistical significance was defined as *P < 0.05 and **P < 0.01. For tissue microarray USP18 immunohistochemical scores were grouped based on comparison to normal muscle as 1+ (low) or 2-3+ (retained). This scoring system is previously described [32]. For primary soft tissue tumors only, the method of Kaplan and Meier was used to assess outcomes in relation to USP18 expression. Univariable and multivariable Cox proportional hazards regression models were used to estimate associations between USP18 expression and overall survival (OS), disease-specific survival (DSS) and time to first metastasis, with alpha of 0.05 considered as significant.

## Results

### FVB-Usp18 knockout mice develop leiomyosarcomas

**Fig. 1:**
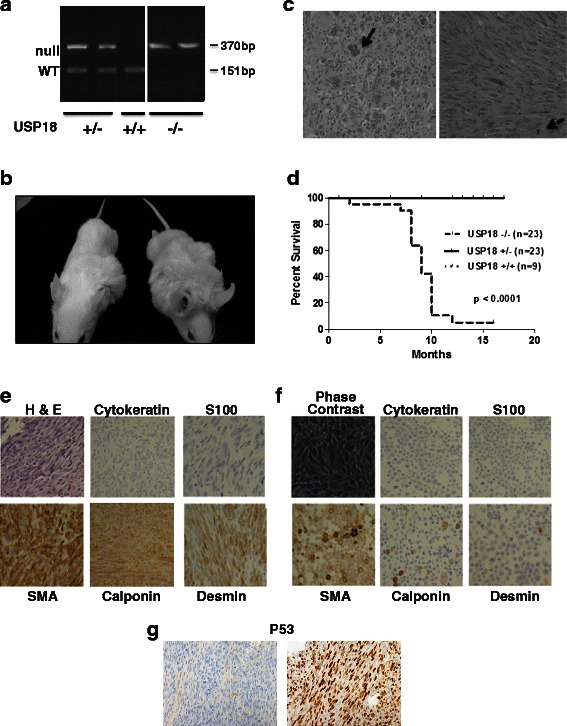
FVB-USP18 knockout mice develop sarcomas. **a**: PCR genotyping assays of USP18−/−, USP18+/−, and USP18+/+ genomic DNA (null allele, 370bp and wild-type allele 151bp). **b**: USP18 null mice with tumors. **c**: H & E staining of representative sarcomas. The solid arrow indicates a multinucleate giant cell. The spindle cell characteristic of the sarcomas is depicted in the right panel and the hatched arrow indicates a mitotic cell. **d**: Kaplan-Meier curve is displayed depicting the times when sarcoma development necessitated euthanasia in USP18−/− mice versus USP18+/+ and USP18+/− mice, respectively. USP18 null mice significantly developed sarcomas (P < 0.0001) while no sarcomas were seen in wild-type or heterozygous mice. **e**: H & E staining of a representative murine leiomyosarcoma in a USP18 −/− mouse is displayed along with the expression profiles for S100, cytokeratin (AE1/AE3), desmin, calponin and SMA (smooth muscle actin). **f**: A representative leiomyosarcoma cell line (KHC-2) was also examined for the same markers displayed in panel E. A phase-contrast image of this cell line is provided. **g**: The P53 immunostaining is of two USP18−/− murine leiomyosarcomas, one positive for nuclear P53 and one negative

USP18 knockout mice on an FVB background were generated and their genotypes confirmed by PCR assays (Fig. 1a). About 80% of FVB-USP18 knockout mice developed clinically-detectable subcutaneous tumors in the trunk or neck regions (Fig. 1b and c). This was in contrast to wild-type and heterozygous USP18 mice where no such tumors were detected (Fig. 1c). In some USP18 knockout mice, dystrophic calcifications appeared with or without tumor development (Additional file 1: Figure S1). H & E staining of formalin-fixed sections of these murine tumors revealed anaplastic tumors with numerous mitotic bodies and a spindle-like morphology (Fig. 1d). Some of the tumors had giant cells (Fig. 1d). The dystrophic calcification could be a precursor lesion for these tumors. Calcification is often seen in leiomyomas, which are benign smooth muscle cell malignancies, but has also been seen in leiomyosarcomas [33].

The sarcomas occurred in USP18 null mice as early as 2 months of age (Table 1). Younger mice (less than 6 months old) tended to exhibit more dystrophic calcifications unlike the gross tumor development in mice older than 6 months (Additional file 1: Figure S1A). Most of the USP18 knockout mice exhibited palpable sarcomas at ages from 6 months to 12 months (Table 1 and Fig. 1b). While there were more male than female mice displayed in Table 1, this was not due to a sex-linked difference in sarcoma development. The percentage of mice that developed sarcomas was similar for males and females.

**Table 1:** Molecular pathology of the murine sarcomas

Mouse # (M/F)	Age (months)	S100	Cytokeratin	SMA	Desmin	Calponin	p53
1 (M)	9	0	0	3+	2+	focal-1+	3+
2 (M)	10	0	rare 2+	3+	2+	3+	0
3 (M)	8	0	rare 2+	3+	3+	0	1+
4 (M)	10	Rare nuclei	rare 1+	3+	2+	2+	1+
5 (M)	9	0	0	3+	3	focal-1+	0
6 (F)	7	0	0	2+	0	NA^*^	0
7 (F)	9	0	0	3+	rare 2+	3+	3+
8 (M)	10	0	0	2+	1+	focal-1+	3+
9 (F)	10	0	0	2+	3+	0	0
10 (M)	9	0	rare 1+	2+	3+	2+	2+
11 (M)	8	0	0	3+	3+	2+	1+
12 (M)	2	0	0	1+	1+	focal-2+	0
13 (M)	9	0	0	2+	2+	focal-1+	3+
KHC-1	N/A	0	0	3+	2+	focal-2+	×
KHC-1	N/A	0	0	3+	2+	2+	×

This Table provides scoring for the different marker antibodies used to diagnose the histopathology of these sarcomas. The markers include S100, cytokeratin (AE1/AE3), smooth muscle actin (SMA), desmin, calponin and p53. All staining was diffuse unless otherwise noted. N/A refers to not applicable. The abbreviations M and F refer to male and female, respectively

NA^*^ is Not Evaluable and x is Not Done

To further characterize these sarcomas, histopathological analyses were performed. These sarcomas were of mesenchymal origin because they were typically negative for the epithelial marker cytokeratin (Table 1). S100, a marker for melanomas and some neural-derived tumors was absent in every sarcoma except for one with rare nuclear staining. Of the mesenchymal-specific cell markers, all 13 of the tumors stained diffusely positive for smooth muscle actin. For desmin, 12 of 13 stained positively with 1 negative and 1 with rare staining. Calponin staining was the lowest of the three mesenchymal markers examined and it exhibited diffusely positive staining in 5 tumors and focal staining in another 5; 2 tumors stained negatively and another tumor was not evaluable (Table 1). All the tumors analyzed were classified as high-grade leiomyosarcomas and these sarcomas exhibited necrosis. A representative sarcoma and sarcoma derived cell line are shown in Fig. 1e and f. For the cell line shown in Fig. 1f and for some of the other sarcomas there was some heterogeneity of staining with calponin and desmin.

Mice lacking the tumor suppressor P53 spontaneously develop tumors, specifically lymphomas and sarcomas [34]. Studies of clinical leiomyosarcomas have shown frequent deregulation of P53 [12]. In this study, 5 of 13 sarcomas showed abundant staining for P53 indicating stabilization and deregulation of this protein. Figure 1 shows a tumor with abundant nuclear P53 and another that was negative for P53 staining (Fig. 1g).

### Restoration of USP18 expression in USP18 null leiomyosarcoma cell lines

**Fig. 2:**
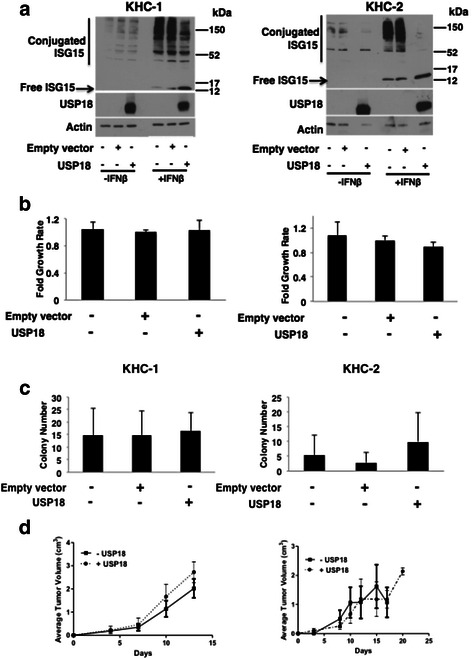
Reconstitution of USP18 in USP18−/− sarcoma cell lines did not reverse tumorigenesis. **a**: Stable transfection of human USP18 restored USP18 deconjugase activity in the USP18 knockout KHC-1 and KHC-2 leiomyosarcoma cell lines. The ISG15 conjugates were more prominent after 24 h treatment with IFNβ. **b**: Monolayer growth assays of empty vector versus USP18 reconstituted cells over 72 h expressed as fold difference versus control cells. **c**: Anchorage-independent growth for KHC-1 and KHC-2 cell lines, with or without USP18 reconstitution. **d**: Tumorigenesis of KHC-1 and KHC-2 cells in athymic mice in USP18 reconstituted cells versus vector control cells. Error bars represent standard deviation

Leiomyosarcoma cell lines were derived from two different mice with sarcomas and were designated as KHC-1 and KHC-2, respectively (Fig. 2a). Loss of the USP18 gene in each cell line was confirmed by PCR assay genotyping. To determine if restoring USP18 activity reversed tumorigenicity in these cell lines, each was retrovirally-transduced with either human USP18 or an empty vector. USP18 functional reconstitution was demonstrated by decreased ISG15 conjugates in response to IFNβ treatment versus parental and empty vector control cells (Fig. 2a).

There was no statistically significant difference in monolayer or soft agar growth assays between KHC-1 and KHC-2 cells independently stably transfected with an empty vector or the USP18 expression vector (Fig. 2b and c). When these leiomyosarcoma cell lines were each transplanted into athymic mice (*n* = 4), palpable tumors arose within 2 to 3 days of subcutaneous implantation. Tumors grew at a similar rate and reached their predetermined study endpoint diameter of 15mm within 10–20 days (data not shown). Engrafted tumors were harvested from athymic mice and USP18 expression in the USP18 stably transfected tumor-forming cells was detected (Additional file 1: Figure S1B). Tumor histology was similar to the sarcomas in USP18 null mice. There was no statistically significant difference observed in tumor growth between cells transduced with an empty vector versus those with restored USP18 activity (Fig. 2d).

This experiment was repeated in immunocompetent FVB/N mice and there was no difference in tumor growth (or immune infiltrates) detected between cells transplanted with restored USP18 activity and controls (*n* = 4). Tumor onset and immune infiltrates were similar to that observed in athymic mice (data not shown). Hence, an intact immune system did not affect murine leiomyosarcomas growth. While loss of USP18 preceded sarcoma formation, these sarcoma cell lines did not depend on loss of USP18 expression for survival, growth or tumorigenicity.

### USP18 null leiomyosarcoma cell lines are aneuploid and overexpress MYC

**Fig. 3:**
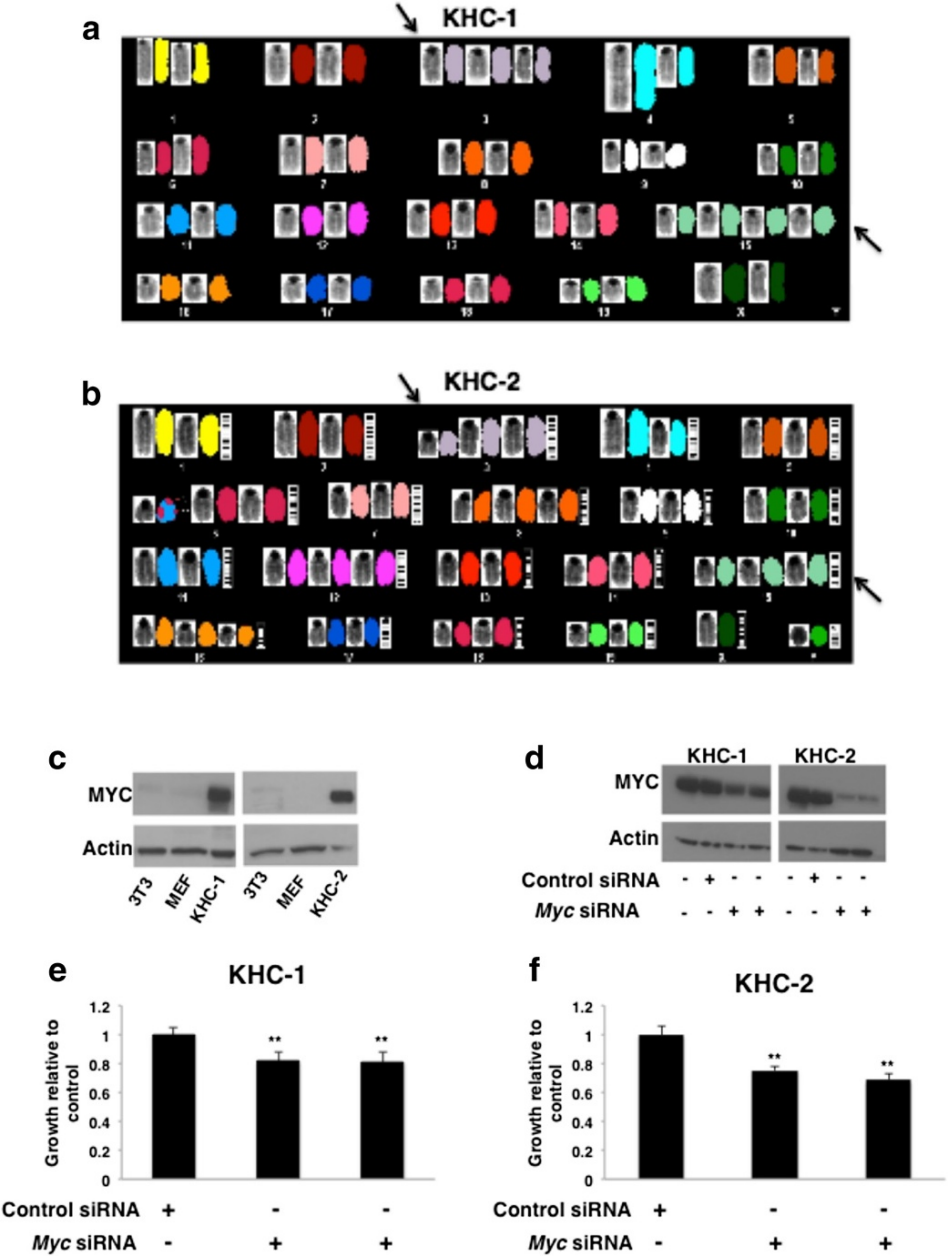
Leiomyosarcoma cell lines are aneuploid and overexpress MYC. **a** and **b**: Cytogenetic analyses showed that both KHC-1 and KHC-2 had polysomy of chromosomes 3 and 15 (*black arrows*). **c**: Immunoblot analysis of MYC protein as shown for KHC-1 and KHC-2 cell lines as compared to NIH-3T3L1 and mouse embryonic fibroblast (MEF) cells. **d**: Immunoblot analysis of MYC levels validated knockdown by two independent siRNAs in KHC-1 and KHC-2 cells. **e** and **f**: Analysis of cell growth in KHC-1 and KHC-2 cells transfected with MYC or control siRNAs

Since leiomyosarcomas can exhibit aneuploidy [10, 11], cytogenetic analyses were performed on KHC-1 and KHC-2 cell lines. Standard karyotyping by G-banding and spectral karyotyping (SKY) were used to characterize the cells and both were aneuploid (Fig. 3a and b). The KHC-1 cell line had trisomy 3, tetrasomy 15, deletions of chromosomes 3 and 4 and a derivative chromosome 9. KHC-2 cells showed trisomy 3, 12 and 15, trisomies with derivatives on 8 and 16, and a translocation of chromosomes 6 and 11 (Fig. 3b). Chromosome 15 contains the loci for *Myc* (orthologous to a chromosome 8 region in humans). Chromosome 15 is also the site of two proto-oncogenes of the Wnt family, Wnt1 and Wnt10b [35]. Aneuploidy is frequent in clinical leiomyosarcomas and the chromosomal sites shown here are homologous to those altered in human leiomyosarcomas [15, 16].

Given that both cell lines exhibited polysomy in chromosome 15, which carries the *Myc* locus (Fig. 3a and b), MYC protein levels were examined in KHC-1 and KHC-2 cells. Compared to wild-type mouse embryonic fibroblasts (MEFs) and NIH-3T3L1 cells, MYC protein levels were higher in the leiomyosarcoma cell lines (Fig. 3c). To determine the effect of MYC repression in these cell lines, two different MYC siRNAs were individually transfected into each cell line and growth effects were compared with controls after 72 h. Knockdown of MYC protein in each cell line was confirmed (Fig. 3d) with MYC protein levels decreased to between 12–27 % and 25–35 % of controls in KHC-1 cells and KHC-2 cells, respectively, as determined by densitometry from 3 independent experiments. Reduced MYC expression modestly, yet significantly inhibited growth of both cell lines (Fig. 3e and f).

### IFNβ and doxorubicin treatments of murine and human leiomyosarcoma cell lines

**Fig. 4:**
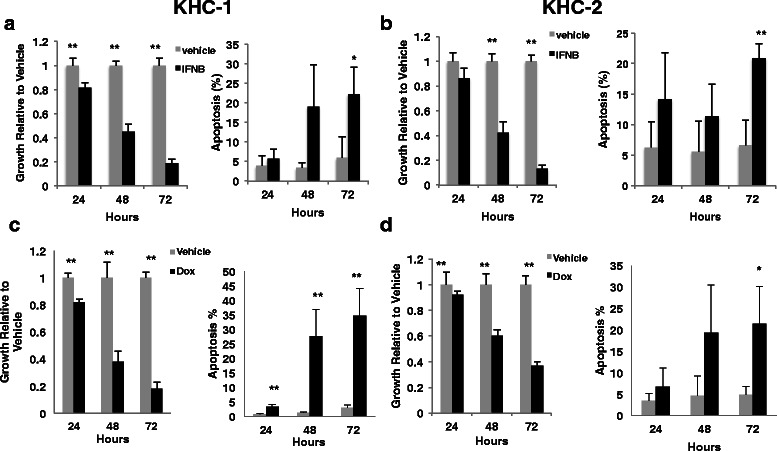
USP18−/− leiomyosarcoma cell lines are sensitive to IFNβ and clinically-achievable concentrations of doxorubicin. **a** and **b**: Cell growth and apoptosis analysis of KHC-1 and KHC-2 cells treated with IFNβ (500 units/mL). **c** and **d**: Cell growth and apoptosis analysis of KHC-1 and KHC-2 cells treated with 0.2 μM doxorubicin (Dox). Analyses were done at 24, 48 and 72 h post-treatment for both doxorubicin and IFNβ

USP18 knockout mice on a C57Bl/6 background were hypersensitive to type I IFN [23]. Given this, we sought to treat USP18 null sarcoma cell lines with IFNβ to determine if they exhibited type I IFN hypersensitivity. Growth inhibition occurred 24, 48 and 72 h after IFNβ treatment, with the most prominent inhibition at 72 h (Fig. 4a and b, left panels). Both of these leiomyosarcoma lines exhibited apoptosis after IFNβ treatments (Fig. 4a and b, right panels). Independent treatments of a human leiomyosarcoma cell line with identical IFNβ and doxorubicin concentrations conferred modest growth inhibition (Additional file 1: Figure.S3). Due to the slower growth characteristics of this cell line, it cannot be assumed that the USP18 null cells are hypersinsitive to IFNβ. Treatment of the murine USP18 null cell lines with restored USP18 activity did not alter IFNβ response. This indicated that USP18 levels did not affect IFNβ sensitivity in these cells when grown in vitro (Additional file 1: Figure.S4).

Immunoblot analyses of the murine cell lines uncovered constitutive phosphorylation of STAT3, which is oncogenic in other systems [36]. With development of inhibitors of the upstream kinase Janus kinase 2 (JAK2), it is possible to target this pathway. KHC1 and KHC2 cells were each growth inhibited by the JAK2-STAT3 pathway inhibitor JSI-124 (Additional file 1: Figure. S2) at doses that inhibited JAK2-STAT3 signal transduction (Additional file 1: Figure. S2C). These sarcoma cell lines were also sensitive to clinically achievable doxorubicin dosages that caused decreased proliferation and increased apoptosis in these cells (Fig. 4c and d). Thus, these new murine leiomyosarcoma cell lines established antineoplastic activity of known and new agents for treatment of leiomyosarcomas.

### USP18 expression in human leiomyosarcomas

**Fig. 5:**
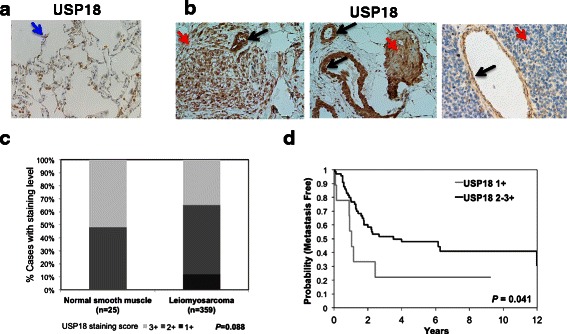
Immunohistochemical analyses in human leiomyosarcoma cases and normal vascular smooth muscle and outcomes analysis. **a**: USP18 staining of normal lung tissue. Blue arrow indicates a USP18 positive macrophage. **b**: USP18 staining of clinical leiomyosarcoma with adjacent normal smooth muscle tissue. Abundant USP18 expression in vascular smooth muscle cells is highlighted with black arrows. Red arrows indicate leiomyosarcoma. **c**: Relative levels of USP18 in normal smooth muscle and in a series of leiomyosarcoma. **d**: Time to development of first metastasis for 89 primary uterine and soft tissue leiomyosarcoma patients

To establish the translational research relevance of these pre-clinical findings, USP18 immunohistochemical studies were performed in 21 different human leiomyosarcomas (Additional file 1: Table S2). Specimen site is indicated since samples were from a mix of primary tumors, metastases and tumors that had recurred. A representative immunohistochemical analysis of USP18 levels in histopathologically normal human lung and in clinical leiomyosarcomas is displayed in Fig. 5a and b. Findings revealed prominent USP18 immunostaining in lung macrophages (Fig. 5a) and also in histopathologically normal vascular smooth muscle cells with reduced expression relative to the adjacent leiomyosarcoma (Fig. 5b). Some leiomyosarcomas expressed USP18 levels approaching that of histologically normal smooth muscle (Fig. 5b center panel). Vascular smooth muscle is the cell of origin for some leiomyosarcomas [37, 38].

To extend this analysis, USP18 levels were examined in a human leiomyosarcoma tissue microarray. Compared to normal tissues where USP18 staining was high, (vascular smooth muscle cells served as a control), there was a sub-group of leiomyosarcomas with reduced USP18 immunostaining (Fig. 5c). Clinical data were available for 89 primary uterine and soft tissue leiomyosarcomas. In univariable Cox proportional hazards analysis, retained USP18 expression was associated with prolonged time to first metastasis (HR 0.4543, 95 % CI 0.2107–0.9795, *p* = 0.0441) (Fig. 5d). USP18 levels were not predictive of overall survival (OS) (*p* = 0.72) or disease free survival (DSS) (*p* = 0.3371) (data not shown). Multivariate analysis was performed including factors shown in prior analyses as clinically prognostic in leiomyosarcoma [32]. In the current analysis, USP18 retained expression predicted longer time to metastasis (*p* = 0.0213).

## Discussion

Leiomyosarcomas have complex karyotypes and treatment options are limited [39, 40]. Clinically-relevant murine leiomyosarcoma models should help develop innovative therapies for these sarcomas. Few soft-tissue murine models of leiomyosarcoma exist. Inactivation of PTEN in smooth muscle cells produced small mice with smooth muscle hyperplasia affecting blood vessels and the urinary and intestinal tracts, but not in the uterus [41]. Expression of the T antigens of the SV40 early region resulted in a narrow tissue-specific distribution of tumors. All female animals developed large uterine leiomyosarcomas by 3 months and most males exhibited enlarged seminal vesicles from smooth muscle hyperplasia [42]. In an experiment to specifically inactivate BRCA1, P53 and Rb in murine ovarian epithelial cells, tumors arose instead from adjacent smooth muscle and were diagnosed as leiomyosarcomas [43]. Thus, distinct genetic changes can cause leiomyosarcomas to arise from different primary sites.

The 3-MCA-carcinogen murine fibrosarcoma model provided insights into tumor immunology and cancer immunoediting. Mice with specific immune defects are particularly susceptible to chemical-induced carcinogenesis. Perforin-1, IFN-γ, STAT-1 and T cells each contribute to reducing 3-MCA-induced tumor formation and growth [44]. The immune system can drive tumors into a dormant state, which in turn affects immunoediting and tumorigenicity, as reviewed [44].

Defects in IFN signaling can augment tumorigenesis in the 3-MCA-sarcoma model; evidence from USP18 null mice identified an amplified IFN response as compared to wild-type mice, which is thought to provide a less permissive environment for tumors [45]. To directly explore the role of IFN signaling in leiomyosarcoma development it would prove informative in the future to cross the USP18 null mice with mice lacking either IFN or the IFN receptor (IFNGR1). However, transplantation of KHC-1 cells into immunocompentent mice did not result in a prominent immune cell response (data not shown), arguing against a driving role for the immune system in suppressing development of these leiomyosarcomas.

Abundant USP18 immunostaining in vascular smooth muscle cells implied that sarcomas seen in these mice could be of vascular origin. USP18 expression levels in clinical leiomyosarcoma varied widely with some staining at levels comparable to vascular smooth muscle and some with much lower levels (Fig. 5a and Additional file 1: Table S2). A significant association between USP18 levels and time to onset of metastasis was determined confirming a link between USP18 levels and disease progression, as shown in Fig. 5.

Sarcomas were not seen in the original report of USP18 null mice likely because this was in the C57/Bl6 and 129 mixed strain background. These mice developed neurological symptoms that reduced their survival to less than 20 weeks of age [21]. In marked contrast, USP18 null mice in this study had been bred onto the FVB/N strain background and rarely exhibited neurological symptoms.

USP18 null mice developed leiomyosarcomas with complex karyotypes and distinct genetic alterations reminiscent of human leiomyosarcomas. Common alterations included MYC overexpression and P53 stabilization. The genetic variability of leiomyosarcoma makes it essential to have different models in which to test antineoplastic drug treatments. Loss of PTEN sensitized leiomyosarcoma cells to rapamycin treatment [41], however tumors with different driver mutations will likely have different drug sensitivities.

Loss of USP18 activity is associated with type 1 IFN hypersensitivity [21]. Both murine USP18 null and human leiomyosarcoma cell lines described here responded independently to doxorubicin and type 1 IFN treatments. Activating the IFN pathway either directly with IFN or with an IFN-inducing agent might target leiomyosarcomas. Murine leiomyosarcoma cell lines also exhibited constitutive activation of phosphorylated STAT3 (pSTAT3). JAK2-STAT3 inhibitor treatment of these sarcoma cells led to downregulation of pSTAT3 and growth inhibition, indicating this type of inhibitor presents a potential treatment for leiomyosarcomas (Additional file 1: Figure. S2).

USP18 is the major deconjugase for ISG15, a type 1 IFN-regulated ubiquitin-like protein modifier [18]. Decrease in USP18 has not been previously associated with sarcoma development. Conversely, USP18 repression has been associated with antineoplastic effects in lung and kidney cancers and in acute promyelocytic leukemia [26–28]. Immunohistochemical analysis of human tissues showed high levels of USP18 expression in normal vascular smooth muscle cells from which these tumors are likely derived [38]. IFN is known to stimulate vascular smooth muscle cell proliferation via PI3K and mTOR signaling [46]. The IFN-hypersensitive environment found in USP18 null mice might deregulate proliferation of these vascular cells, initiating leiomyosarcoma formation. This possibility is consistent with the leiomyosarcoma formation seen when PI3K/mTOR signaling is inappropriately activated in smooth muscle cells by deletion of the negative regulator, PTEN [41].

## Conclusions

This study reports a new murine model of spontaneous leiomyosarcoma with associated transplantable cell lines. The tumors exhibited many features of human leiomyosarcomas including aneuploidy, MYC amplification and P53 stabilization. Sarcomas rapidly developed after transplantation of murine leiomyosarcoma cell lines into recipient mice, making this a tractable model for testing innovative leiomyosarcoma therapies. Taken together, these models can provide insights into both leiomyosarcoma biology and therapy.

## Additional files

Additional file 1: Table S1. Antibodies used for immunohistochemistry studies. **Table S2.** Immunohistochemical staining of USP18 in clinical leiomyosarcoma samples. **Figure S1.** A: Dystrophic calcifications in USP18 null mice. A representative image of a USP18-/- mouse is shown. B: KHC-2 cells with reconstituted USP18 expression maintained expression for the duration of growth in mice. Immunoblot analysis of protein isolated from 4 control and 4 USP18 overexpressing independent orthotopic sarcomas harvested from mice. The immunoblot showed representative analysis of KHC-2 cells and this finding was also seen in KHC-1 cells (data not shown). **Figure S2.** USP18 null leiomyosarcoma cell lines are sensitive to treatment with the JAK2-STAT3 inhibitor, JSI-124. A: Immunoblot analysis of pSTAT3, STAT3, CDK4 and USP18 levels in KHC-1 cells with and without stably restored USP18 activity. B: Growth analysis of KHC-1 cells with JAK2-STAT3 inhibitor, JSI-124. Similar effects were seen in KHC-2 cells (data not shown). Validation of JSI-124 repression of JAK2-STAT3 pathway C: Immunoblot analyses of phosphorylated JAK2 (pJAK2), JAK2, and actin with relative level of pJAK2/JAK2 calculated relative to control. D: Immunoblot analysis of phosphorylated STAT3 (pSTAT3), STAT3, cyclin D1 and actin levels. **Figure S3.** USP18 null leiomyosarcoma cell line KHC-1 and human leiomyosarcoma cell line SK-LMS-1 growth in response to interferon-β (500Units/ml IFNB) or doxycycline (0.2μM Dox) treatment over 3 days. Results expressed as fold relative to vehicle treated cells. Each experiment was performed in triplicate 3 separate times. **Figure S4.** USP18 null leiomyosarcoma cell line KHC-1 with restored USP18 expression did not affect response to IFNb (500Units/ml). Results expressed as fold relative to vehicle treated cells. (N.S. = not significant). (PPTX 2131 kb)
Additional file 2: **Mice Null for the Deubiquitinase USP18 Spontaneously Develop Leiomyosarcoma.** (DOCX 115 kb)
