# Supplementary material for: Mice null for the deubiquitinase USP18 spontaneously develop leiomyosarcomas
Source: BMC Cancer. 2015 Nov 10;15:886. doi: 10.1186/s12885-015-1883-8 (PMC4640382; doi:10.1186/s12885-015-1883-8)
Supplement: Additional file 1: Table S1. — Antibodies used for immunohistochemistry studies. Table S2. Immunohistochemical staining of USP18 in clinical leiomyosarcoma samples. Figure S1. A: Dystrophic calcifications in USP18 null mice. A representative image of a USP18-/- mouse is shown. B: KHC-2 cells with reconstituted USP18 expression maintained expression for the duration of growth in mice. Immunoblot analysis of protein isolated from 4 control and 4 USP18 overexpressing independent orthotopic sarcomas harvested from mice. The immunoblot showed representative analysis of KHC-2 cells and this finding was also seen in KHC-1 cells (data not shown). Figure S2. USP18 null leiomyosarcoma cell lines are sensitive to treatment with the JAK2-STAT3 inhibitor, JSI-124. A: Immunoblot analysis of pSTAT3, STAT3, CDK4 and USP18 levels in KHC-1 cells with and without stably restored USP18 activity. B: Growth analysis of KHC-1 cells with JAK2-STAT3 inhibitor, JSI-124. Similar effects were seen in KHC-2 cells (data not shown). Validation of JSI-124 repression of JAK2-STAT3 pathway C: Immunoblot analyses of phosphorylated JAK2 (pJAK2), JAK2, and actin with relative level of pJAK2/JAK2 calculated relative to control. D: Immunoblot analysis of phosphorylated STAT3 (pSTAT3), STAT3, cyclin D1 and actin levels. Figure S3. USP18 null leiomyosarcoma cell line KHC-1 and human leiomyosarcoma cell line SK-LMS-1 growth in response to interferon-β (500Units/ml IFNB) or doxycycline (0.2μM Dox) treatment over 3 days. Results expressed as fold relative to vehicle treated cells. Each experiment was performed in triplicate 3 separate times. Figure S4. USP18 null leiomyosarcoma cell line KHC-1 with restored USP18 expression did not affect response to IFNb (500Units/ml). Results expressed as fold relative to vehicle treated cells. (N.S. = not significant). (PPTX 2131 kb) [file 12885_2015_1883_MOESM1_ESM.pptx]

## Slide 1
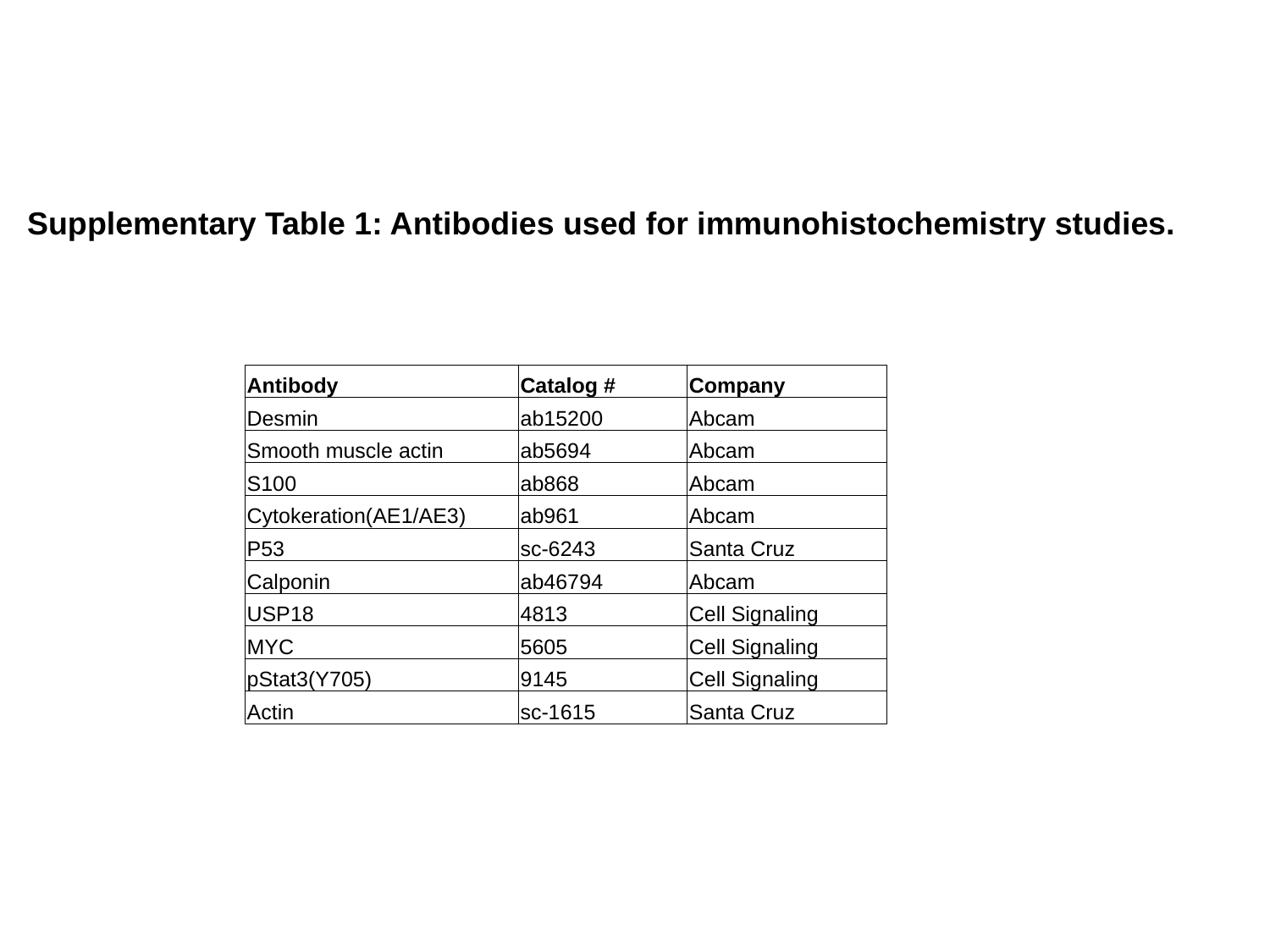

Supplementary Table 1: Antibodies used for immunohistochemistry studies.
| Antibody | Catalog # | Company |
| --- | --- | --- |
| Desmin | ab15200 | Abcam |
| Smooth muscle actin | ab5694 | Abcam |
| S100 | ab868 | Abcam |
| Cytokeration(AE1/AE3) | ab961 | Abcam |
| P53 | sc-6243 | Santa Cruz |
| Calponin | ab46794 | Abcam |
| USP18 | 4813 | Cell Signaling |
| MYC | 5605 | Cell Signaling |
| pStat3(Y705) | 9145 | Cell Signaling |
| Actin | sc-1615 | Santa Cruz |

## Slide 2
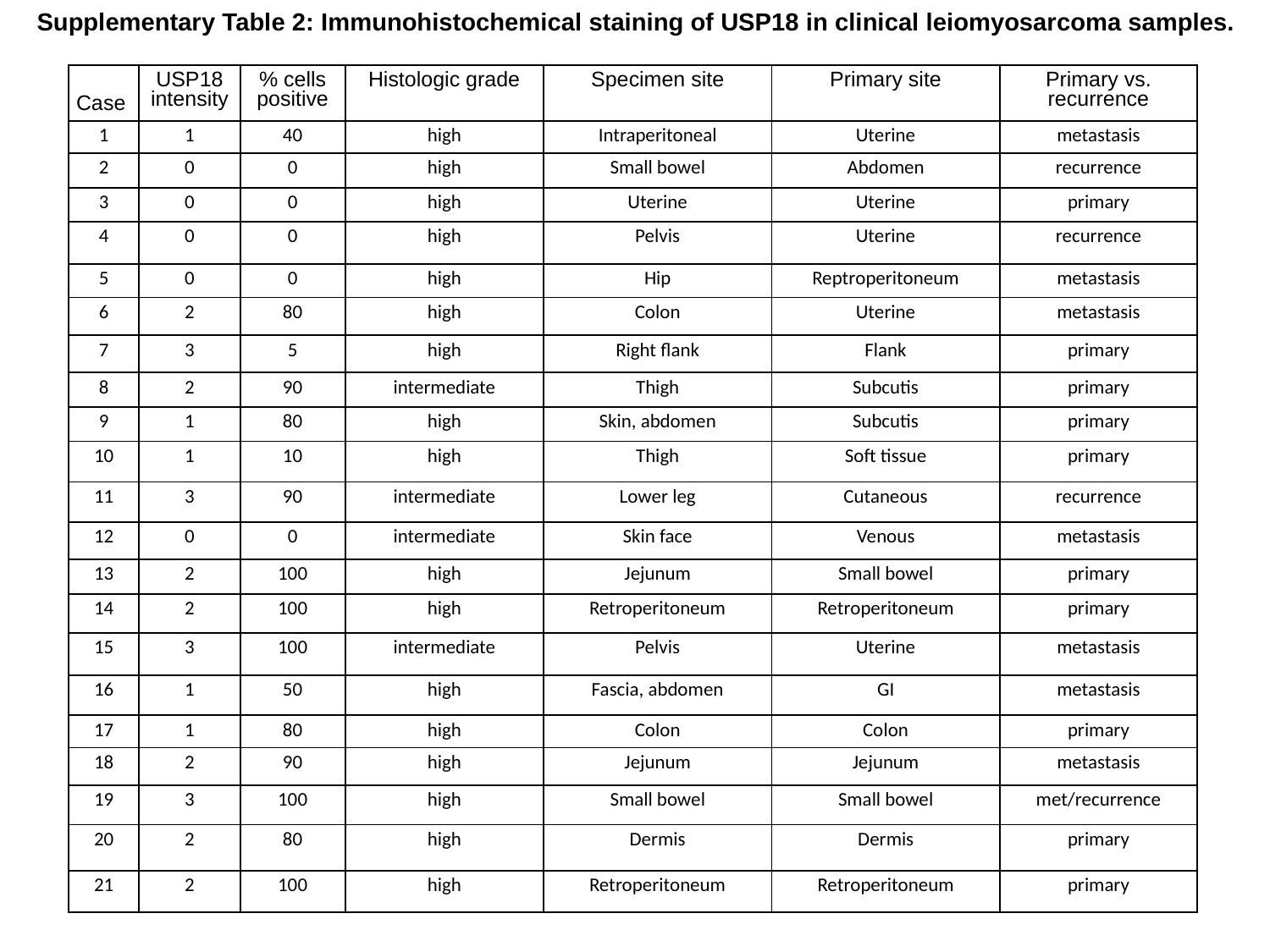

Supplementary Table 2: Immunohistochemical staining of USP18 in clinical leiomyosarcoma samples.
| Case | USP18 intensity | % cells positive | Histologic grade | Specimen site | Primary site | Primary vs. recurrence |
| --- | --- | --- | --- | --- | --- | --- |
| 1 | 1 | 40 | high | Intraperitoneal | Uterine | metastasis |
| 2 | 0 | 0 | high | Small bowel | Abdomen | recurrence |
| 3 | 0 | 0 | high | Uterine | Uterine | primary |
| 4 | 0 | 0 | high | Pelvis | Uterine | recurrence |
| 5 | 0 | 0 | high | Hip | Reptroperitoneum | metastasis |
| 6 | 2 | 80 | high | Colon | Uterine | metastasis |
| 7 | 3 | 5 | high | Right flank | Flank | primary |
| 8 | 2 | 90 | intermediate | Thigh | Subcutis | primary |
| 9 | 1 | 80 | high | Skin, abdomen | Subcutis | primary |
| 10 | 1 | 10 | high | Thigh | Soft tissue | primary |
| 11 | 3 | 90 | intermediate | Lower leg | Cutaneous | recurrence |
| 12 | 0 | 0 | intermediate | Skin face | Venous | metastasis |
| 13 | 2 | 100 | high | Jejunum | Small bowel | primary |
| 14 | 2 | 100 | high | Retroperitoneum | Retroperitoneum | primary |
| 15 | 3 | 100 | intermediate | Pelvis | Uterine | metastasis |
| 16 | 1 | 50 | high | Fascia, abdomen | GI | metastasis |
| 17 | 1 | 80 | high | Colon | Colon | primary |
| 18 | 2 | 90 | high | Jejunum | Jejunum | metastasis |
| 19 | 3 | 100 | high | Small bowel | Small bowel | met/recurrence |
| 20 | 2 | 80 | high | Dermis | Dermis | primary |
| 21 | 2 | 100 | high | Retroperitoneum | Retroperitoneum | primary |

## Slide 3
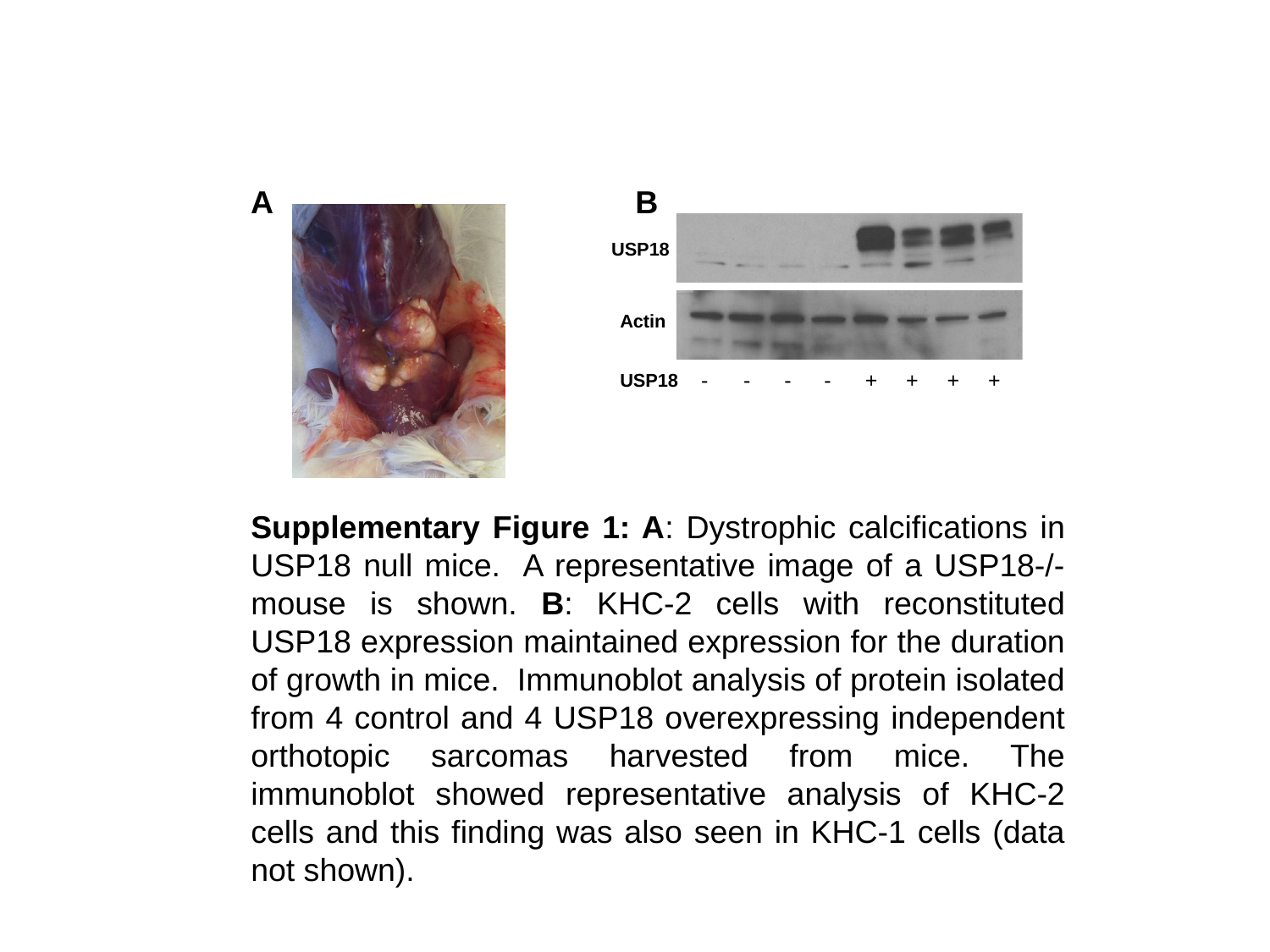

A
B
USP18
Actin
| - | - | - | - | + | + | + | + |
| --- | --- | --- | --- | --- | --- | --- | --- |
USP18
Supplementary Figure 1: A: Dystrophic calcifications in USP18 null mice. A representative image of a USP18-/- mouse is shown. B: KHC-2 cells with reconstituted USP18 expression maintained expression for the duration of growth in mice. Immunoblot analysis of protein isolated from 4 control and 4 USP18 overexpressing independent orthotopic sarcomas harvested from mice. The immunoblot showed representative analysis of KHC-2 cells and this finding was also seen in KHC-1 cells (data not shown).

## Slide 4
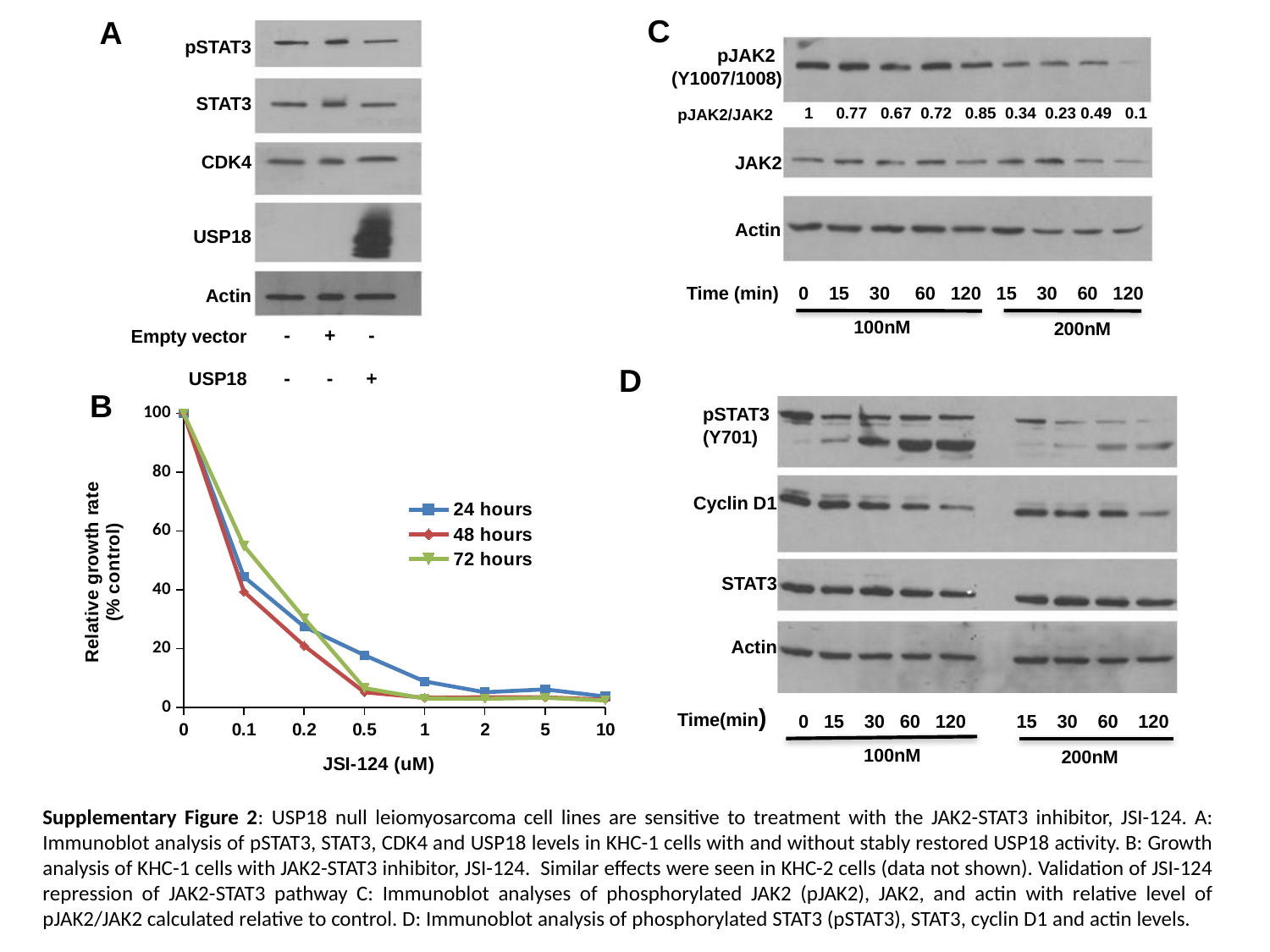

C
A
pSTAT3
 pJAK2
(Y1007/1008)
STAT3
0.77 0.67 0.72 0.85 0.34 0.23 0.49 0.1
pJAK2/JAK2
JAK2
CDK4
Actin
USP18
Time (min)
0 15 30 60 120 15 30 60 120
Actin
100nM
200nM
| - | + | - |
| --- | --- | --- |
| - | - | + |
Empty vector
D
USP18
B
### Chart
| Category | | | |
|---|---|---|---|
| 0.0 | 100.0 | 100.0 | 100.0 |
| 0.1 | 44.43204868154157 | 39.36609402050872 | 54.93975903614457 |
| 0.2 | 27.4290060851927 | 20.94819549873485 | 30.32339885859227 |
| 0.5 | 17.74340770791075 | 5.145825009988005 | 6.535193405199747 |
| 1.0 | 8.8184584178499 | 3.265414835530696 | 2.999365884590996 |
| 2.0 | 5.131845841784989 | 3.433213477160741 | 2.911857958148383 |
| 5.0 | 6.105476673427964 | 3.402583566387002 | 3.250475586556754 |
| 10.0 | 3.682555780933063 | 2.646157943800772 | 2.348763474952441 |pSTAT3
(Y701)
Cyclin D1
STAT3
Actin
Time(min)
0 15 30 60 120 15 30 60 120
100nM
200nM
Supplementary Figure 2: USP18 null leiomyosarcoma cell lines are sensitive to treatment with the JAK2-STAT3 inhibitor, JSI-124. A: Immunoblot analysis of pSTAT3, STAT3, CDK4 and USP18 levels in KHC-1 cells with and without stably restored USP18 activity. B: Growth analysis of KHC-1 cells with JAK2-STAT3 inhibitor, JSI-124. Similar effects were seen in KHC-2 cells (data not shown). Validation of JSI-124 repression of JAK2-STAT3 pathway C: Immunoblot analyses of phosphorylated JAK2 (pJAK2), JAK2, and actin with relative level of pJAK2/JAK2 calculated relative to control. D: Immunoblot analysis of phosphorylated STAT3 (pSTAT3), STAT3, cyclin D1 and actin levels.

## Slide 5
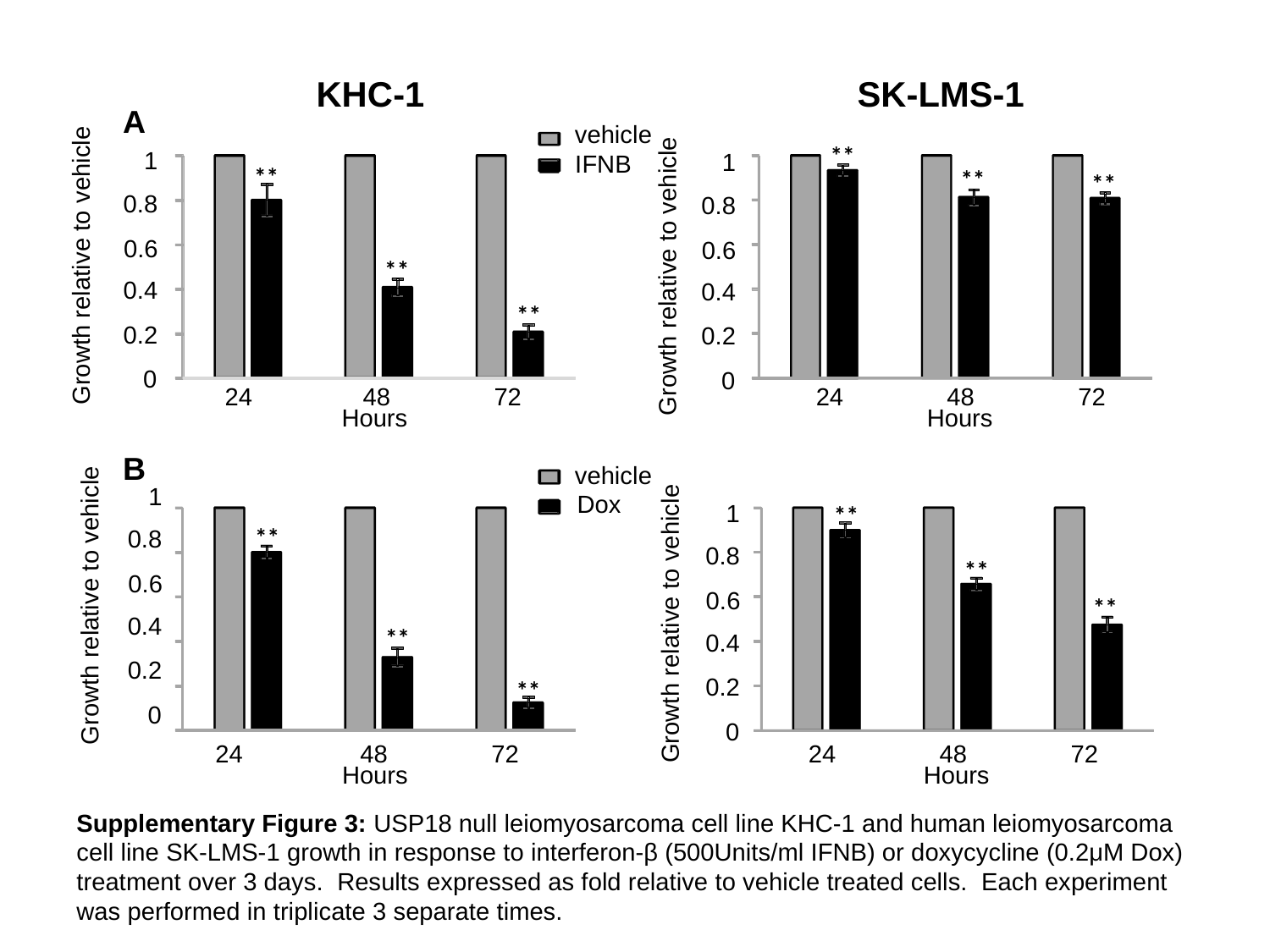

KHC-1
SK-LMS-1
A
vehicle
IFNB
**
1
0.8
0.6
0.4
0.2
0
1
0.8
0.6
0.4
0.2
0
**
**
**
Growth relative to vehicle
Growth relative to vehicle
**
**
24
 48
 72
24
48
72
Hours
Hours
B
vehicle
Dox
1
0.8
0.6
0.4
0.2
0
**
1
0.8
0.6
0.4
0.2
0
**
**
Growth relative to vehicle
**
Growth relative to vehicle
**
**
24
 48
 72
24
48
72
Hours
Hours
Supplementary Figure 3: USP18 null leiomyosarcoma cell line KHC-1 and human leiomyosarcoma cell line SK-LMS-1 growth in response to interferon-β (500Units/ml IFNB) or doxycycline (0.2μM Dox) treatment over 3 days. Results expressed as fold relative to vehicle treated cells. Each experiment
was performed in triplicate 3 separate times.

## Slide 6
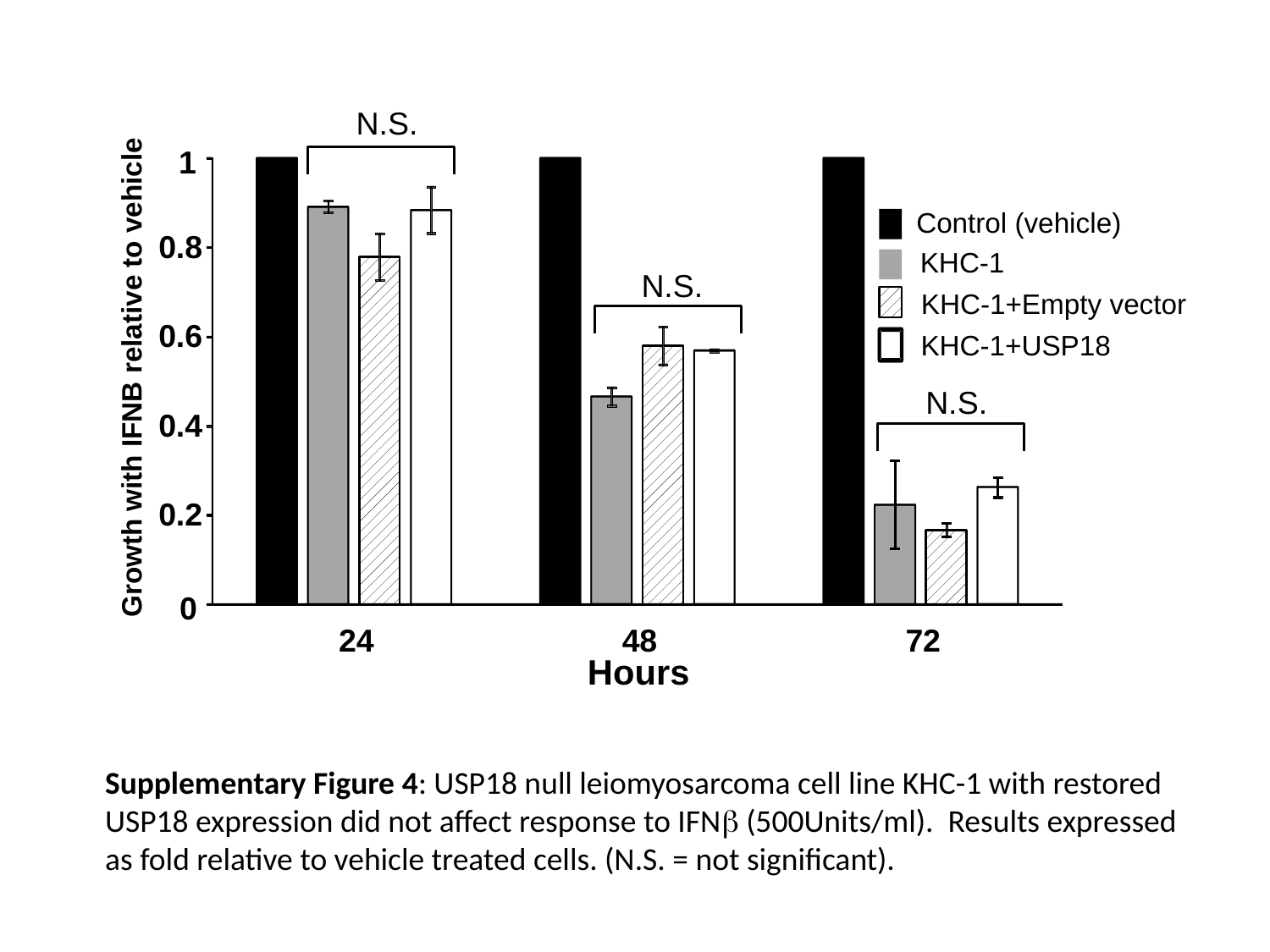

N.S.
1
Control (vehicle)
0.8
KHC-1
N.S.
KHC-1+Empty vector
0.6
KHC-1+USP18
Growth with IFNB relative to vehicle
N.S.
0.4
0.2
0
24
48
72
Hours
Supplementary Figure 4: USP18 null leiomyosarcoma cell line KHC-1 with restored USP18 expression did not affect response to IFNb (500Units/ml). Results expressed as fold relative to vehicle treated cells. (N.S. = not significant).
