# Supplementary material for: Mice null for the deubiquitinase USP18 spontaneously develop leiomyosarcomas
Source: BMC Cancer. 2015 Nov 10;15:886. doi: 10.1186/s12885-015-1883-8 (PMC4640382; doi:10.1186/s12885-015-1883-8)
Supplement: Additional file 2: — Mice Null for the Deubiquitinase USP18 Spontaneously Develop Leiomyosarcoma. (DOCX 115 kb) [file 12885_2015_1883_MOESM2_ESM.docx]

**Supplemental Methods**

**Mice Null for the Deubiquitinase USP18 Spontaneously Develop Leiomyosarcoma**

**Patients and tissues**

Leiomyosarcoma specimens were obtained at Dartmouth Hitchcock Medical Center and M.D. Anderson Cancer Center using protocols approved by the Institutional Review Boards of Dartmouth Hitchcock Medical Center and M.D. Anderson Cancer Center. Patients provided written informed consent.

**Immunohistochemistry**

Sarcomas were sectioned and air-dried at room temperature. Staining was performed using the Leica Bond Max Automated Stainer. The automated protocol included baking slides for 30 minutes and dewaxing using Bond Dewax solution (Leica Biosystems). Bond Epitope Retrieval was used for antigen retrieval. Primary antibody binding was visualized using either a Bond Refine Detection kit (Leica Biosystems) or a mouse-on-mouse HRP-Polymer kit (Biocare) with DAB chromogen (Novocastra, Leica Biosystems) and hematoxylin counterstain. Antibodies used for immunohistochemistry are listed in Supplementary Table 1. The immunohistochemical assays were independently reviewed by two pathologists (P.J.H.) and (V.M.) and concordant results are displayed.

**Tissue microarrays**

Previously described uterine and non-uterine leiomyosarcoma tissue microarrays were utilized (1). Briefly, tissue microarrays (TMA) contain 384 leiomyosarcoma specimens from 257 patients on 8 TMA blocks. In addition, the TMAs contain as controls; 10 samples of normal muscularis propria, 10 normal myometrium, 5 myometrium from patients with uterine leiomyosarcoma (ULMS), 5 uterine leiomyomas, and 5 esophageal leiomyomas. All samples are cored in duplicate (1.2 mm cores).

**Chromosomal analyses**

The metaphases were arrested by incubation with Colcemid (KaryoMax® Colcemid Solution, Invitrogen, 10μg/ml) overnight prior to harvest. Cells were collected and treated with hypotonic solution (0.075M KCl) for 15 minutes at 37°C and fixed with a methanol - acetic acid mixture (3:1vol/vol). Slides were prepared and aged overnight for use in spectral karyotyping (SKY) analysis and G-banding. Chromosomes were stained with a trypsin-Giemsa staining technique (2). Analyses were performed under an Axioplan 2 (Zeiss) microscope coupled with a CCD camera (ASI); images were captured with Band View 5.5 karyotyping software, (Applied Spectral Imaging Inc.). The karyotype was determined by comparison to the standard ideogram of banding patterns for mouse chromosomes (3). Spectral karyotyping was performed (4).

**Spectral karyotyping**

Spectral karyotyping was performed as previously (4). Metaphases were hybridized with the 21-color mouse SKY paint kit (ASI, Applied Spectral Imaging Inc.) according to manufacturer’s protocol. Hybridization was carried out in a humidity chamber at 37°C for 16 hours. The post-hybridization washing was used as before (4). Detection was carried out following Applied Spectral Imaging’s protocol. Spectral images of the hybridized metaphases were acquired using a SD301 SpectraCube^TM^ system (Applied Spectral Imaging Inc.) mounted on an epifluorescence microscope Axioplan 2 (Zeiss). Images were analyzed using Spectral Imaging 6.0 acquisition software (Applied Spectral Imaging). G-banding was simulated by electronic inversion of DAPI counterstaining. A minimum of 10 mitoses of comparable staining intensity and quality was examined per cell line and were compared for chromosomal abnormality.

**References**

1. Demicco E.G., Boland G.M., Brewer Savannah K.J., Lusby K., Young E.D., Ingram D., Watson K.L., Bailey M., Guo X., Hornick J.L., van de Rijn M., Wang W.L., Torres K.E., Lev D., Lazar A.J. (2015) Progressive loss of myogenic differentiation in leiomyosarcoma has prognostic value. **Histopathology**. 66: 627-38.

2. Schreck, R.R., Disteche, C. (1994). Chromosome Banding Techniques. **Current Protocols in Human Genetics**, Unit 4.2

3. Nesbitt MN, Francke U. (1973). A system of nomenclature for band patterns of mouse chromosomes. **Chromosoma** 41:145-158.

4. Schrock E, du Manoir S, Veldman T, et al. (1996) Multicolor spectral karyotyping of human chromosomes. **Science**. 273:494–497.
